# Association between central blood pressure, arterial stiffness, and mild cognitive impairment

**DOI:** 10.1186/s40885-016-0058-5

**Published:** 2017-01-15

**Authors:** R. Suleman, R. Padwal, P. Hamilton, A. Senthilselvan, K. Alagiakrishnan

**Affiliations:** 1 Department of Medicine, Division of General Internal Medicine, University of Alberta, Edmonton, AB Canada; 2 School of Public Health, University of Alberta, Edmonton, AB Canada; 3 Department of Medicine, Division of Geriatric Medicine, University of Alberta, Edmonton, Canada; 4 University of Alberta Hospital, B146, Clinical Sciences Building, 8440-112 Street, Edmonton, T6G 2G3 Canada

**Keywords:** Blood pressure, Augmentation pressure, Augmentation index, Arterial stiffness, Elderly, Cognitive impairment

## Abstract

**Background:**

To determine the relationship between central blood pressure (CBP) indices and mild cognitive impairment (MCI) in adults over the age of 50.

**Methods:**

A cross-sectional study conducted using a non-invasive SphygmoCor XCEL device. CBP indices and brachial blood pressure were measured in 50 inpatients and outpatients. MCI was assessed using the Montreal Cognitive Assessment (MoCA) instrument and by the European Consortium Criteria (ECC).

**Results:**

Seventy-six percent of subjects had hypertension, and 52% were diagnosed as having MCI using the ECC. No significant association was found between any of the measured blood pressure variables and global cognition. A significant relationship was observed between augmentation index (AI) and abnormal clock-drawing (*p* = 0.04) and language (*p* = 0.02), and between pulse pressure amplification (PPA) and language (*p* = 0.03).

**Conclusion:**

CBP indices like AI and PPA, which are markers of vascular stiffness, are associated with poor executive function and language cognitive domain deficits.

## Background

High blood pressure may exert negative effects on the brain and contribute to cognitive decline. Hypertension is seen in 7% of adults aged 18–39 years, 33% of adults aged 40–59 years and 55% of Medicare beneficiaries [1, 2]. A number of cohort and population studies have shown an association between hypertension and cognitive impairment/dementia and these studies point out that hypertension seen in early stages of life can cause cognitive impairment or dementia [3–5]. Blood pressure variability as measured by 24-h non-invasive BP monitoring has been associated with cognitive decline [6]. There is also evidence that lowering blood pressure in middle-age can prevent dementia in later life and hypertension is one of the preventable causes of cognitive decline [7, 8]. Conversely, evidence from large randomized control trials demonstrated an inconsistent relationship between antihypertensives and cognitive decline [9].

Although blood pressure is typically measured peripherally over the brachial artery, central blood pressure (CBP) measurements, including aortic blood pressure, have shown stronger associations with end-organ damage and may be of greater clinical use. CBP can be measured non-invasively using validated cuff- based techniques. Recent studies indicate that the interaction of vascular stiffness and wave dynamics in the large arteries is an important mediator of blood pressure elevation with aging. Pressure waves travelling from the left ventricle to the aorta and subsequently to the peripheral arteries are reflected at the arterial junction, resulting in wave retropulsion, which augments pressure in the aortic root. A recent, prospective, observational study showed a temporal relationship between vascular stiffness, blood pressure elevation and progression to hypertension [10].

Arterial stiffness of the large vessels influences the pumping of blood from the heart and is an independent predictor of future cardiovascular events beyond blood pressure alone. Arterial stiffness measures both structural (arterial media) and dynamic arterial components (tone of the smooth muscle in the arterial media). Different blood pressure indices are used to measure arterial stiffness (carotid-femoral pulse wave velocity (cfPWV)) and its associated hemodynamic sequelae with wave reflections (Pulse Pressure Amplification(PPA) and Augmentation Index(AI)). These blood pressure indices improve incremental cardiovascular risk stratification and prediction of future CV outcomes [11]. cfPWV is the common measure of arterial stiffness (meters/second). AI is an alternate parameter, representing a ratio measuring the relationship of forward and backward travelling waves in the central aorta. PPA reflects the magnitude of blood pressure augmentation between the central and peripheral arteries [12].

The association of CBP indices and cognitive decline has not been widely studied. Therefore, the objective of this study was to determine the relationship between central blood pressure (CBP) indices and the cognitive function of adults over the age of 50 years. This study may give further insights into the association between blood pressure and cognitive deterioration.

## Methods

In this cross-sectional study, using a non-invasive SphygmoCor XCEL device (AtCor Medical), CBP indices (blood pressure, augmentation pressure, augmentation index, pulse pressure amplification, and pulse wave velocity) and traditional brachial blood pressure (BBP) were measured in 50 inpatients and outpatients at the University of Alberta Hospital and Kaye Edmonton Clinic. Non-English speaking subjects and those with a history of dementia, blindness, depression, anxiety, mental illness, substance abuse and chronic pain were excluded. Subjects were also excluded when they were medically unstable or terminally ill, under isolation precautions or unable to give informed consent. Data on demographic variables and vascular risk factors including hypertension, diabetes and hyperlipidemia was obtained. Information on history of transient ischemic attacks (TIA’s), strokes and anti-hypertensive medications was also obtained.

### BP measurements

Blood pressure measurements were obtained after patients had been resting in a supine position for at least 10 min. Both brachial and central BP indices were measured at the beginning and again at the end of the assessment, with each brachial BP measurement resulting from the average of two back-to-back readings. Central BP indices were generated non-invasively from a brachial artery waveform using the generalized transfer function of the Sphygmocor XCEL software. Pulse wave velocity was calculated using the same device, by measurement of the distance and pulse transit time between the carotid and femoral arteries (with waveforms captured through carotid applanation tonometry and a femoral BP cuff, respectively).

### Cognitive tests

Cognitive testing was done using MoCA [13] and European Consortium Criteria (ECC) [14]. MoCA measures seven cognitive domains including executive function and abstraction. MoCA has excellent test-retest reliability, and the internal consistency on the items in MoCA is 0.83. A MoCA score of 26 or less was considered to signify mild cognitive impairment (MCI). Participants were classified as having MCI using the following definitions operationalized for this study. A MCI diagnosis was based on the screening test MoCA (<26 points)(normal or abnormal) as well as using the European Consortium Criteria(present or absent). MCI was diagnosed according to the European Consortium on Alzheimer’s disease criteria as the currently available standard test to diagnose MCI [14]. Functional information on daily activities was collected using Katz Basic Activities of Daily living (BADL) and Lawton Instrumental Activities of Daily Living (IADL) questionnaires [15, 16]. The Consortium criteria include (1) cognitive complaints from the patients or their families, (2) a decline in cognitive functioning relative to previous abilities during the past year reported by the patient or informant, (3) cognitive disorders as evidenced by clinical evaluation (impairment in memory or in another cognitive domain, which in this study was assessed by MoCA), (4) absence of major repercussions on daily life (in this study, was measured by Katz BADL and Lawton IADL), and (5) absence of dementia (in this study, dementia was ruled out by using DSM IV criteria [17]). Since depression can cause cognitive deficits, it was ruled out by using DSM IV criteria for depression [17].

### Statistical analysis

Descriptive statistics was performed on all demographic and clinical parameters. The prevalence of cognitive impairment was calculated. Baseline patient characteristics were reported as percentages for categorical variables and means and standard deviations for continuous ones. Logistic regression models were constructed to examine the independent association between peripheral or central BP with the MoCA score (normal or abnormal) as well as the individual cognitive domains (normal or abnormal), and the ECC diagnosis of MCI (present or absent) separately. All statistical analyses were performed using SPSS, with two-sided p-values with statistical significance set at 0.05.

Ethics approval was obtained from the University of Alberta Research Ethics Board. Written informed consent was obtained from all the participants in the study.

## Results

The average age (± SD) of the subjects was 70 ± 12 years and the average body mass index was 29 ± 6 kg/m^2^, with 56% being female, 76% having hypertension, and 42% being smokers (Table 1). Of the 38 patients on anti-hypertensive medication, 21% used an ACE-I or ARB alone, 16% used an ACE-I/ARB and CCB, 13% used an ACE-I/ARB and beta-blocker, 8% used an ACE-I/ARB and thiazide diuretic, and 8% used an ACE-I/ARB, CCB, and thiazide diuretic. The remaining 34% of patients with hypertension used a variety of other single agents, or combination of agents.

**Table 1:** Baseline demographics

Characteristics	Patient Sample (*n* = 50) Mean (SD) or No (%)
Age (years): mean (SD)	70 (12)
Body mass index (kg/m^2^): mean (SD)	29 (6)
Female	28 (56%)
Hypertension	38 (76%)
Dyslipidemia	29 (58%)
Past or current smoker	21 (42%)
History of TIA	2 (4%)
History of stroke	5 (10%)
Diabetes	20 (40%)
Type I	1 (2%)
Type II	19 (38%)
MoCA score: mean (SD)	21 (5)
Diagnosed with MCI	26 (52%)

Fifty-two percent of patients were diagnosed as having MCI using the ECC. No significant relationship was seen between any of the measured blood pressure variables (central or peripheral) and MCI. Mean blood pressures levels between normal and abnormal clock drawing and language domains are shown in Table 2. When individual cognitive domains were analysed with logistic regression analysis adjusted for age and sex, there was a significant relationship between the augmentation index (AI) and clock-drawing completion (OR: 1.08; 95% CI 1.00–1.16, *p* = 0.04) and language (OR: 1.14; 95% CI: 1.02–1.27; *p* = 0.02). As shown in Table 3, a significant relationship was also seen between pulse pressure amplification and the language sub domain (OR: 0.36; 95% CI 0.14–0.91; *p* = 0.03).

**Table 2:** Mean blood pressure levels in patients with normal and abnormal clock-drawing and language scores^a^

Index	Clock-Drawing		Language	
	Normal (mean ± SD) *n* = 17	Abnormal (mean ± SD) *n* = 32	Normal (mean ± SD) *n* = 8	Abnormal (mean ± SD) *n* = 41
Average brachial systolic blood pressure	142.7 ± 24.8	140.7 ± 18.8	146.2 ± 31.5	140.5 ± 18.5
Average brachial diastolic blood pressure	77.6 ± 13.5	79.3 ± 10.5	84.9 ± 15.7	77.5 ± 10.3
Average brachial pulse pressure	65.1 ± 17.4	61.5 ± 14.4	61.3 ± 18.9	63.0 ± 14.9
Average mean arterial pressure	97.0 ± 16.9	99.2 ± 12.5	103.4 ± 20.7	97.5 ± 12.5
Average central systolic blood pressure	126.2 ± 21.6	127.5 ± 17.3	128.7 ± 27.2	126.8 ± 17.0
Average central diastolic blood pressure	79.1 ± 13.4	80.3 ± 10.3	86.0 ± 15.7	78.7 ± 10.1
Average central pulse pressure	47.1 ± 14.8	47.2 ± 12.5	42.7 ± 14.7	48.0 ± 13.0
Average central augmentation pressure	10.1 ± 8.1	14.4 ± 7.1	7.8 ± 6.9†	13.9 ± 7.5†
Average central augmentation index	18.1 ± 14.1‡	29.3 ± 9.8‡	15.3 ± 12.8†	27.4 ± 11.7†
Average pulse pressure amplification	1.4 ± 0.2†	1.3 ± 0.1†	1.5 ± 0.1†	1.3 ± 0.1†
Pulse wave velocity	9.0 ± 2.0	9.3 ± 1.8	9.1 ± 1.5	9.1 ± 2.0

^a^Mann-Whitney U tests were conducted on the blood pressure indices in those with normal vs. abnormal clock-drawing scores, and normal vs. abnormal language scores. Significant results are noted below

†*p* < 0.05

‡*p* < 0.01

**Table 3:** Association between blood pressure indices and measures of cognitive function-results from logistic regression^a^

Index	Association with Abnormal Clock-Drawing		Association with Abnormal Language Score	
	Odds ratio (95% CI)	*p*-value	Odds ratio (95% CI)	*p*-value
Average brachial systolic blood pressure	0.98 (0.95, 1.02)	0.31	0.93 (0.85, 1.01)	0.08
Average brachial diastolic blood pressure	1.00 (0.95, 1.06)	0.92	0.93 (0.86, 1.01)	0.09
Average brachial pulse pressure	0.96 (0.92, 1.01)	0.14	0.94 (0.87, 1.02)	0.12
Average mean arterial pressure	1.00 (0.95, 1.04)	0.90	0.94 (0.87, 1.02)	0.11
Average central systolic blood pressure	0.99 (0.95, 1.03)	0.55	0.95 (0.89, 1.01)	0.12
Average central diastolic blood pressure	1.00 (0.95, 1.06)	1.00	0.93 (0.85, 1.01)	0.09
Average central pulse pressure	0.97 (0.92, 1.03)	0.37	0.97 (0.88, 1.06)	0.48
Average central augmentation pressure	1.06 (0.95, 1.18)	0.29	1.17 (0.98, 1.41)	0.09
Average central augmentation index	1.08 (1.00, 1.16)	0.04	1.14 (1.02, 1.27)	0.02
Average pulse pressure amplification (for 0.1 unit increase)	0.60 (0.34,1.07)	0.08	0.36 (0.14, 0.91)	0.03
Pulse wave velocity	1.06 (0.72, 1.55)	0.78	0.53 (0.20, 1.41)	0.20

^a^Adjusted for age and sex

Measurements of blood pressure indices were generally reliable, with good intra-rater reliability indicated by the intra-class correlation coefficients ranging from 0.92 to 0.97 (Table 4).

**Table 4:** Intra-rater reliability between repeated measures of blood pressure indices

Index	Intraclass Correlation Coefficient (95% CI)
Brachial systolic blood pressure	0.97 (0.95, 0.98)
Brachial diastolic blood pressure	0.97 (0.94, 0.98)
Brachial pulse pressure	0.96 (0.92, 0.98)
Mean arterial pressure	0.97 (0.94, 0.98)
Central systolic blood pressure	0.97 (0.96, 0.99)
Central diastolic blood pressure	0.96 (0.93, 0.98)
Central pulse pressure	0.97 (0.94, 0.98)
Central augmentation pressure	0.94 (0.89, 0.96)
Central augmentation index	0.92 (0.85, 0.95)

## Discussion

Hypertension is the most important modifiable risk factor for the development and progression of cognitive decline. Cognitive impairment can range from simple memory loss to deficits in executive function, manifesting as difficulties with planning and organizing tasks, managing time, self-care, and decreased processing speed [18]. In the Canadian Study of Health and Aging (CSHA), executive function was the earliest-affected cognitive domain and preceded the development of dementia [19]. CBP may be more relevant to the study of cognitive impairment than PBP since the blood is delivered to the brain through the large central arteries. It has been shown that in hypertensive subjects, target organ damage is mediated not only by steady pressure, but also by pulsatile hemodynamics and blood flow [20]. A systematic review showed arterial stiffness is associated with cerebral small vessel disease and decreased cognitive function [21]. In this study, we analyzed the associations between central and peripheral blood pressure indices and both global and domain-specific cognitive function, adjusted for age and sex. Standard measures of brachial and central BP were not found to be associated with global cognitive decline or with individual domains of cognition. In this study, we did not further adjust for other co-morbidities such as hypertension or diabetes status because these conditions are causes of arterial stiffness and mediate the co-linear relationship between blood pressure, arterial stiffness and outcomes.

The clinical evaluation of arterial stiffness and its pulsatile hemodynamics is complex. A few studies have shown a relationship between CBP parameters such as PP and PWV and indices of cognitive function [22–26]. Among the CBP indices we evaluated in this study, AI and PPA were found to be significantly associated with deficits in certain cognitive domains. A lower PPA, reflecting accelerated arterial aging, was associated with abnormal language function, and a higher AI was associated with both abnormal language and executive function. Different CBP indices like cfPWV, PPA, and AI may be measuring different aspects of arterial mechanics, and there is some evidence that AI may be a better marker for cardiovascular outcomes. For instance, AI has previously been shown to distinguish the effects of different BP medications, when brachial blood pressure and cfPWV were not able to [27]. Although Mitchell et al. [28] did not find a relationship between AI and cognitive function, their study only evaluated adults aged 69 and older, whereas our study included adults over the age of 50. With progressive aging, central impedance increases faster than peripheral impedance and AI can consequently become dissociated from cfPWV and other markers of arterial stiffness [28]. In addition, with older adults marked stiffening of the aorta may reduce wave reflection at the interface between aorta and proximal large arteries, which result in reduced local and global wave reflections which may contribute to the dissociation between measures of aortic stiffness and AI [28].

Tsao et al. in their study showed similar results to our study in that cfPWV was not associated with cognitive decline [29]. This lack of association may be attributable to differences in cognitive tests (MoCA was used in this study, whereas MMSE was used in other studies). Small sample size may have reduced the effect size and our sensitivity to detect association between all blood pressure indices and cognitive outcomes. Finally, carotid-femoral pulse wave velocity (cfPWV) seems to mainly measure arterial stiffness, whereas pulse pressure amplification (PPA) and Augmentation Index (AI) mainly measures the hemodynamic wave reflections from the arterial wall. So these BP indices may be measuring different aspects of the arterial wall mechanics as well as arterial flow dynamics and hence the difference seen in this study.

A strength of this study is its use of the MoCA for cognitive assessment, which is a more sensitive test for assessing mild cognitive impairment than the Mini-Mental State Examination (MMSE), and which also incorporates an assessment of executive function. We also employed the standardized Consortium Criteria for the diagnosis of MCI. To our knowledge, this is the first study to show an association between high AI and abnormal language and executive function.

Limitations of this study include its cross-sectional nature, which shows only association and not causation, and the small sample size. We did not calculate the sample size. However, we observed significant relationships between two independent variables (augmentation index and pulse pressure amplification) and two aspects of cognition (clock-drawing and language). Although there was a relationship between these variables and the primary end-point of the study (presence or absence of mild cognitive impairment), it did not reach statistical significance. In addition, as expected augmentation index (mean ± SD) was higher in those with MCI (MCI present 26.27 ± 13.81vs MCI absent 23.40 ± 12.28), and pulse pressure amplification was to be lower in those with MCI (MCI present 1.34 ± 0.13 vs MCI absent 1.37 ± 0.14) indicating that our data fit the experimental model.

## Conclusion

Results from this study suggests that CBP indices like AI and PPA, which are markers of vascular stiffness, wave dynamics and augmentation pressure are associated with possible poor executive function and language cognitive domain deficits. These CBP indices may be sensitive indicators of cognitive decline. Future large studies are needed to clarify the effect of CBP indices on cognitive aging.

## Abbreviations

AI: Augmentation index
BBP: Brachial blood pressure
CBP: Central blood pressure
cfPWV: Carotid femoral pulse wave velocity
ECC: European consortium criteria
Katz BADL: Katz basic activities of daily living
Lawton IADL: Lawton instrumental activities of daily living
MCI: Mild cognitive impairment
MMSE: Mini mental status exam
MoCA: Montreal cognitive assessment
PPA: Pulse pressure amplification
TIA: Transient ischemic attack
